# Psychiatrists’ Understanding and Management of Conversion Disorder: A Bi-National Survey and Comparison with Neurologists

**DOI:** 10.2147/NDT.S256446

**Published:** 2020-08-12

**Authors:** Benjamin Dent, Biba R Stanton, Richard A Kanaan

**Affiliations:** 1 Department of Psychiatry, University of Melbourne, Austin Health, Heidelberg, VIC, Australia; 2 Department of Neurology, King’s College Hospital, London, UK; 3 Department of Neuropsychiatry, South London & Maudsley NHS Trust, London, UK; 4 Institute of Psychiatry, Psychology and Neurosciences, King’s College London, London, UK

**Keywords:** conversion disorder, functional neurological disorder, neuropsychiatrist, attitudes, feigning

## Abstract

**Background:**

A 2011 survey of neurologists’ attitudes to conversion disorder found a tacit acceptance of the psychological model but significant ambivalence around its relationship to feigning. These issues are under increased scrutiny as the DSM-5 revision removed both the requirement for a psychological formulation and the exclusion of feigning from the diagnostic criteria. Whether those attitudes are shared with psychiatrists is unknown.

**Methods:**

An online survey of the Section of Neuropsychiatry, Royal Australian and New Zealand College of Psychiatrists, and the Faculty of Neuropsychiatry, Royal College of Psychiatrists (UK), on their understanding and management of conversion disorder in February 2019. Statistical comparisons are made with our previous survey of Neurologists.

**Results:**

A total of 52 Australian and 131 UK-based members completed the survey which revealed similarities but also clear differences from their neurological colleagues. The psychiatrists strongly endorsed a psychogenic model for conversion disorder, and the conversion model in particular, though many models were employed. They felt a psychiatric assessment was essential to the diagnosis of conversion disorder, and they often disagreed with the diagnosis in neurology referrals of putative conversion disorder. Most felt that a psychiatric formulation was supportive, and many that it was necessary to the diagnosis. They saw feigning as usually present to a degree but were more comfortable with discussing this than neurologists.

**Conclusion:**

Psychiatrists use psychosocial models for conversion disorder and see an overlap with feigning. They believe psychiatrists are essential for the diagnostic process and would not usually support a diagnosis without a psychiatric formulation.

## Introduction

Conversion disorder has long been a controversial disorder for clinicians.^1^ It is traditionally conceived as a psychogenic reaction,^2^ yet it is diagnosed and managed largely by neurologists, who usually feel this is outside of their skill set.^3^ Neurologists are also unsure of the relationship of conversion disorder with feigning, perhaps as a result of their diagnostic approach, which has classically emphasised diagnostic “tricks”.^4^ We conducted a survey of neurologists on their attitudes to understanding and managing the disorder,^5^ which confirmed these impressions: though they accepted psychological models, they were uncomfortable in applying them, and there was considerable overlap with their concept of feigning. Furthermore, though the diagnostic process seemed to require two-steps and two-disciplines – neuropathology exclusion by a neurologist, followed by psychiatric formulation and the exclusion of feigning by a psychiatrist – the neurologists often found psychiatrists unhelpful in this.^6^

In 2013, the 5th revision of DSM^7^ addressed these concerns: the criteria dropped the requirement for psychological formulation (the existence of a preceding stressor) and for the exclusion of feigning – and as the new criteria now rest primarily on the neurological evidence, the diagnosis can now be made by a neurologist alone.^8^ The 11th revision of ICD, while its changes are less radical, also now includes some subtypes of conversion disorder in the neurology section.^9^

Perhaps surprisingly, given these changes, the attitudes of psychiatrists to this have not been systematically assessed. In particular, whether the attitudes and difficulties we identified in neurologists are unique to them or are shared by their psychiatric counterparts is unknown. However, interviews with neuropsychiatrists, the psychiatrists who tend to see conversion disorder most often, suggested that at least some felt that psychiatric formulation was not only possible but essential to the diagnosis,^6^ so that diagnosis by neurologists alone would be inappropriate. We sought to assess the views of psychiatrists on these issues by surveying them using, as far as practicable, the survey previously conducted with neurologists.

## Methods

The survey was initially adapted by RAK from the previous survey,^5^ with questions kept as similar as possible while incorporating the results of previous interviews with neuropsychiatrists on this topic, and accommodating that it was to be surveying psychiatrists rather than neurologists. This was then sent to a panel of three international experts in FND (all consultant neurologists) for quality assurance, with their feedback and other contextual changes incorporated by consensus of the authors. The survey (see Supplementary Data) was then created on Google forms, an online survey application (www.google.com.au/forms/about), and piloted on a sample group of medical students and lay-people to ensure ease of use and understanding. The final survey consisted of 37 questions, largely multiple choice but with some rank-order and free-text questions. These included basic demographic information and details of clinician training, then targeted understanding of conversion disorder and the models employed in clinical practice, as well as attitudes towards the role of neurologists and psychiatrists in patient care. The term “conversion disorder” was used throughout, in line with the previous survey, and no definition was provided – nor was any definition offered for the various psychiatric terms in the survey, to avoid introducing bias: though original definitions for terms such as “psychogenesis”^10^ “dissociation”^11^ and “conversion”^2^ can be found, none of these is universally adopted.

The survey was approved by the Austin Health research ethics committee, and then from the Royal Australian and New Zealand College of Psychiatrists’ (RANZCP) Section of Neuropsychiatry and the United Kingdom (UK) Royal College of Psychiatrists’ (RCPsych) Faculty of Neuropsychiatry, to allow distribution of the survey to their membership lists. Membership of both neuropsychiatry groups is an “opt-in” for fellows and trainees, and does (in Australia) come at a small cost. Membership of either does not require any specific training or experience, so that membership may be defined more by their interest in neuropsychiatry than clinical position or background. An email was sent from each organisation introducing the project and explaining that completing the survey implied consent to participate, and included a hyperlink leading to the online questionnaire which they completed anonymously. Approximately one month after distribution, a reminder email with another link to the survey was sent to all members, including a request not to complete the survey if they had previously done so.

Data collected were entered into and analysed with SPSS Statistics v25 (www.IBM.com). Statistical comparisons to our earlier survey were made where appropriate. P-values represent chi-squared tests unless stated otherwise, with a significance level set at 0.05.

## Results

### Demographics

Emails were sent to the 476 members of the RANZCP Section and 4361 members of the RCPSYCH Faculty of Neuropsychiatry. Thirty-three and 96 members responded, respectively, with a further 19 and 38 members, respectively, responding in the second round. Three responses were excluded from the analysis due to duplication, but all other responses were included in the analysis as they were completed to a satisfactory degree. This gave a completion rate of 52/476 (11%) for Australian and 131/4361 (3%) for UK psychiatrists. Some respondents provided additional comments within the survey or by direct email to the authors, either about the survey design or regarding the nuances of conversion disorder.

The demographics of respondents and their clinical backgrounds are presented in Table 1. Respondents were largely male (63%), between the ages of 31–50, with the great majority (83%) having trained in the United Kingdom and Australia/New Zealand, associated with the sites of survey distribution. Only 19% were employed as neuropsychiatrists. This represents a markedly more female (37% vs 17%, p<0.0001) and slightly younger group (median mid 40s vs high 40s) than the neurologists from our earlier survey, though the psychiatric group included trainees, which the neurologist group did not. Based on their years of psychiatric experience (typical psychiatric training in both jurisdictions being 6 years), 15% of respondents could be expected to still be in training. The majority of respondents (52%) had undertaken some psychodynamic training in addition to the fellowship requirement, and 52% had undertaken some training in neurology (median 3 months), with female respondents significantly less (median 0 vs 3.5 months, p = 0.01, Mann–Whitney *U*-test). The case load of patients presenting with conversion disorder was positively skewed, with a median of four cases seen per year, 5% seeing none and 5% seeing a hundred or more. Eighteen percent reported having exposure to conversion disorder prior to studying, in their friends or family (most frequently their mother) – nearly twice the proportion in neurology (10%, p=0.008). Previous experience of conversion disorder prior to studying medicine was not associated with specific views; however, respondents with prior exposure were more likely to elaborate on “other” options and describe clinical cases which may indicate a deeper interest in the area.

**Table 1:** Demographic and Clinical Experience of Respondents with Percentages Being of Those Answering the Question, Rounded to the Nearest Whole Number

Characteristic	N (%)	
Gender		
Male	115 (63)	
Female	68 (37)	
Age (years)		
<30	7 (4)	
31–40	55 (30)	
41–50	56 (31)	
51–60	39 (21)	
>61	26 (14)	
Country of training		
United Kingdom	115 (63)	
Australia	32 (17)	
India	11 (6)	
New Zealand	6 (3)	
Other	19 (11)	
Specialty of employment		
Liaison psychiatry	40 (22)	
Neuropsychiatry	35 (19)	
Other psychiatry	107 (59)	
Neurology	1 (<1)	
Further psychodynamic training		
Yes	96 (52)	
No	87 (48)	
Experience of conversion before medicine		
None	148 (81)	
In self	0 (0)	
In family member	17 (9)	
In friend	13 (7)	
Combination of the above	2 (1)	
**Characteristic**	**Median**	**Range**
Years in psychiatry	14	1–47
Months in neurology	3	0–60
New CD cases per year	4	0–200

### The Nature of Conversion Disorder

**Figure 1:**
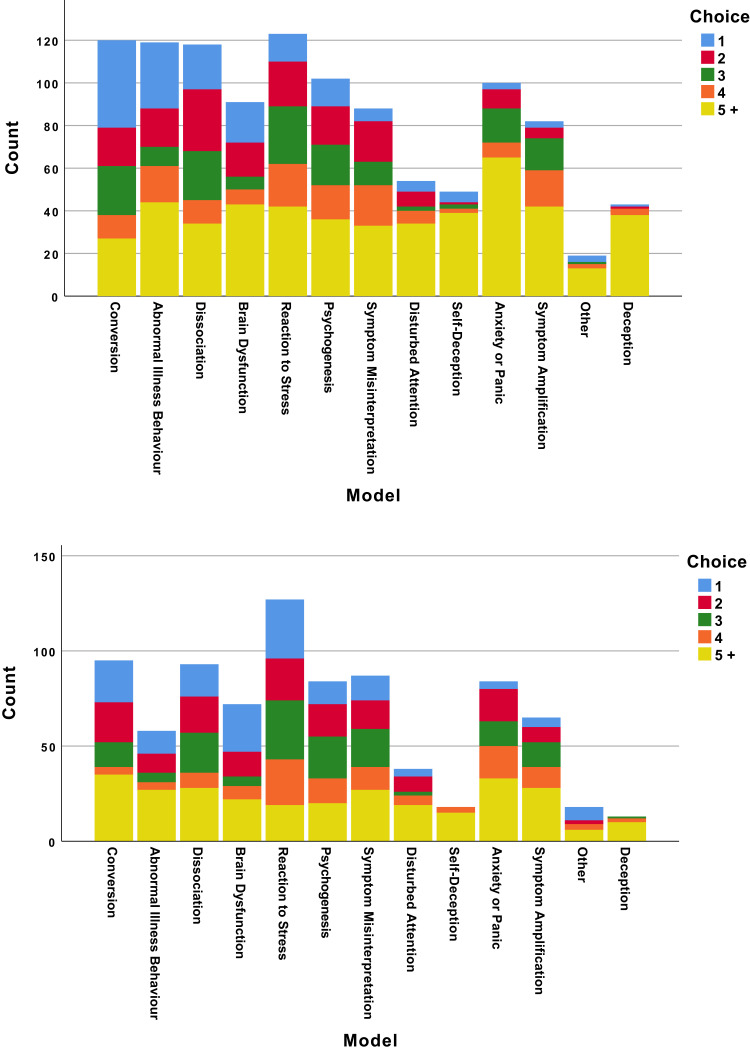
Psychiatrists’ preferred model for understanding (top) and explaining (bottom) conversion disorder. Respondents answered these questions via rank order from 1 to 13, where a characteristic marked “1” was considered most important and “13” to be least important.

Responses regarding the psychiatrists’ attitudes and understanding of conversion disorder are presented in Table 2. The overwhelming view was that psychogenesis was involved (94%), usually (63%) in combination with nervous system dysfunction (this question was not asked in the original survey of neurologists, but came from a Dutch survey,^12^ and our figures differ significantly from the neurologists sampled there (p<0.001, χ^2^=22), with more of our respondents choosing psychogenesis and fewer choosing nervous system dysfunction). Accordingly, though their preferred models for understanding the disorder varied widely (Figure 1), “conversion”, “abnormal illness behaviour”, and “dissociation” were the most commonly accepted. Those with more experience in neurology were less likely to preference “dissociation” (p=0.04, χ^2^=27) or “psychogenesis” (p=0.03, χ^2^=34.6), and female psychiatrists were more inclined towards “psychogenesis” (p=0.02, χ^2^=11.9). Most respondents felt they had a sufficient explanatory model for conversion disorder in general (in contrast with neurologists, most of whom felt psychiatrists did not), and that in an ideal scenario, with sufficient time and patient compliance, they could (50%) usually or always find a psychosocial explanation for a patient’s presentation, though this dropped somewhat (39%) in clinical practice. Yet, 37% said they expected to understand it as a neurological disorder in future, in the same way as Multiple Sclerosis, much higher than the neurologists (p=0.03, χ^2^=7.3).

**Table 2:** Psychiatrists’ Views on the Nature of Conversion Disorder

	N (%)
Do you see the aetiology of conversion disorder as involving:	
Disordered functioning of the nervous system	3 (2)
Psychogenesis	57 (31)
Disordered functioning of the nervous system plus psychogenesis	115 (63)
Feigning	3 (2)
Unknown or other	5 (3)
What is the relationship of conversion disorder to feigning?	
Overlap	102 (56)
Completely distinct	52 (28)
Feigning a subset of conversion	25 (14)
Conversion a subset of feigning	3 (2)
Do you think you have a sufficient model for conversion disorder in general?	
Yes	103 (56)
No	79 (43)
Do you think with enough time and a compliant patient, a psychosocial explanation could be found for a case of conversion disorder?	
Never	0 (0)
Rarely	12 (7)
Often	79 (43)
Usually	83 (45)
Always	9 (5)
How often can you find a psychosocial explanation for a patient’s symptoms in practice?	
Never	1 (<1)
Rarely	14 (8)
Often	96 (52)
Usually	66 (36)
Always	6 (3)
What proportion of your patients referred with unexplained neurological symptoms do you think are feigning?	
None	37 (20)
A few	140 (76)
Many	3 (2)
Most or all of them	1 (<1)
Do you understand conversion disorder to be neurological, in the same way that multiple sclerosis is neurological?	
Yes	14 (8)
Not now, but I expect to one day	67 (37)
No, and I expect I never will	99 (54)

The relationship with feigning differed from that described by neurologists (p=0.001, χ^2^=15.7), with relatively more psychiatrists seeing CD and feigning as overlapping concepts (56%) and relatively fewer seeing them as completely distinct (28%); the proportion of their patients they believed to be feigning was significantly less than the proportion reported by the neurologists (p<0.001, χ^2^=47.5).

Eighty-four respondents described a memorable case, and the same qualitative judgement as in the neurologists’ survey was made by the same author (RAK) about what was distinctive about those cases. Thirty-seven of them fit the categories previously reported, with 19 appearing to be “classic” cases where the formulation made sense of the symptoms, and 16 “dramatic”, where the symptoms or circumstances were unusual, such as a functional blindness; none fit the category of “deception”, significantly fewer than the neurologists (p=0.02, Fisher’s Exact Test), though two did mention apparent secondary gain. Distinctive features for many cases (but not considered categories in the neurologists’ study, and therefore not included in the comparison above) appeared to be the outcomes or responses to treatment, or of particular co-morbidity, notably psychosis and intellectual disability.

### Diagnosing Conversion Disorder

Responses to questions regarding the diagnostic process are presented in Table 3. There was some evidence of wariness of neurologists’ diagnoses, and of confidence in psychiatrists’ own role and skills. Nearly two-thirds (63%) said they felt psychiatrists were essential to the diagnosis, and none that they were unhelpful, a markedly more positive view than neurologists held (p<0.001, χ^2^=67.6). In support of this, 81% said they at least sometimes sent referrals back to the neurologist because the patient did not have a psychiatric disorder (more commonly in the UK (p=0.04, χ^2^=10.3)), and a third (32%) said they often or very often diagnosed the referred patient with a different psychiatric disorder instead. Most felt a neurologist was important to the diagnosis; however, with only a minority (80 participants, 44%) reporting confidence they could make the diagnosis without one often or usually, and none that they could do so always (this confidence, surprisingly, did not vary with experience in neurology, the number of cases seen or the specialty of employment). But they still saw the psychiatric role as necessary: even if the neurologist was certain of the diagnosis, only 24% would agree if they could not formulate the patient’s problem themselves; by contrast, where the neurologist was unsure, the majority of psychiatrists (58%) would make the diagnosis if they could find an explanation for the patients’ problem themselves. None of these attitudes differed with the age or sub-specialty of employment of the psychiatrist, or the number of patients with conversion disorder they saw, though, perhaps surprisingly, those with additional psychodynamic training were less likely to make the diagnosis when the neurologist was unsure (p=0.05, χ^2^=7.9).

**Table 3:** Psychiatrists’ Views on the Diagnosis of Conversion Disorder

	N (%)
If a referring neurologist is sure that a patient has conversion disorder but you cannot explain their symptoms, would you make the diagnosis?	
Yes	44 (24)
Depends on the neurologist	65 (36)
No	73 (40)
What role do psychiatrists have in the diagnosis of conversion disorder?	
Not helpful	0 (0)
Helpful, but not essential	66 (36)
Essential	116 (63)
If a referring neurologist is not sure of the diagnosis, but you can find an explanation, would you make the diagnosis?	
Yes	107 (58)
Depends on the neurologist	39 (21)
No	34 (19)
How often do you send conversion disorder referrals back to neurologists because you do not think the patient has a psychiatric disorder?	
Never	35 (19)
Rarely	115 (63)
Often	30 (16)
Very often	1 (<1)
How often do you diagnose a conversion disorder referral with a different psychiatric disorder instead?	
Never	10 (5)
Rarely	110 (60)
Often	57 (31)
Very often	2 (1)
Would you be confident in diagnosing a case of conversion disorder without assessment by a neurologist?	
Never	26 (14)
Rarely	77 (42)
Often	46 (25)
Usually	34 (19)
Always	0 (0)

### Communicating with Patients and Colleagues

**Figure 2:**
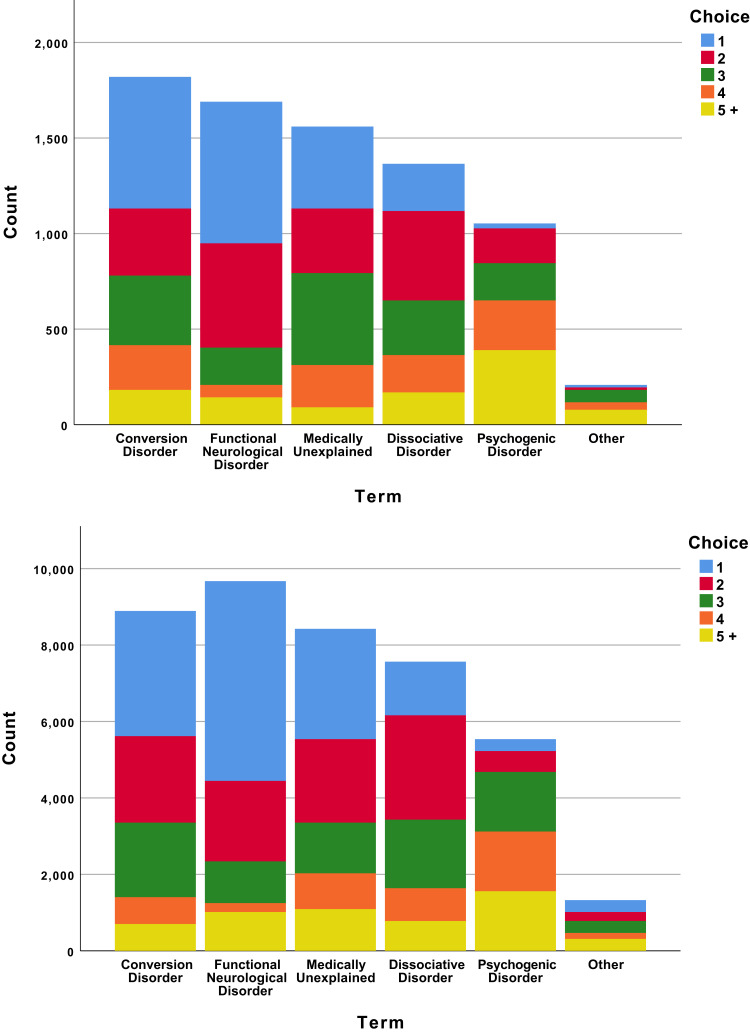
Psychiatrists’ preferred terminology to use with colleagues (top) and patients (bottom). Respondents answered these questions via rank order from 1–6, where a characteristic marked “1” was considered most important and “6” to be least important.

Views of the psychiatrists on communicating with patients and colleagues can be seen in Table 4 and Figures 1 and 2. Figure 1 shows the discordance between models used in understanding and explaining the disorder. The clear favourite as a model for understanding the condition, “conversion” (1st choice for 41, 22%), drops to the third most popular for explaining the condition (1st choice for 22, 12%); while “reaction to stress”, the 5th most popular choice for understanding the condition (1st choice for 13, 7%), is the most popular way to explain it (1st choice for 31, 17%). In terms of terminology, there was less of a shift (Figure 2): though “conversion disorder” was the most used term (by 140, 77%), “functional neurological disorder” was the single most popular choice for speaking to colleagues (1st choice for 57, 31%) and even more popular for speaking to patients (1st choice for 67, 37%).

**Table 4:** Psychiatrists’ Views on Communicating with Patients About Conversion Disorder and Feigning

	N (%)
Do you copy letters about your conversion patients to them?	
Never	27 (15)
Rarely	45 (25)
Often	33 (18)
Usually	41 (22)
Always	33 (18)
Is it important to distinguish feigning from conversion disorder?	
Yes	159 (87)
No	22 (12)
Who should address feigning in your patient?	
Me	45 (25)
General practitioner	4 (2)
Neurologist	5 (3)
Police or some other agency	14 (8)
No-one	10 (5)
Combination of the above	99 (57)
What role do psychiatrists have in the management of conversion disorder?	
Not helpful	0 (0)
Helpful, but not essential	56 (31)
Essential	123 (67)
Do you talk about feigning with patients with unexplained neurology if you suspect it?	
Never	40 (22)
Rarely	80 (44)
Usually	56 (31)
Always	6 (3)
Do you talk about feigning with patients with unexplained neurology if you are sure of it?	
Never	25 (14)
Rarely	56 (31)
Usually	74 (40)
Always	25 (14)
Do you talk about feigning with patients with unexplained neurology when you do not suspect it?	
Never	116 (63)
Rarely	49 (27)
Usually	10 (5)
Always	3 (2)

Only a minority (40%) always or usually sent copies of their letters to patients – a surprisingly low figure, given that this is an expectation for all patients in the UK, though UK psychiatrists did do so more frequently (p<0.001, χ^2^=26.3).

A significant majority (87%) identified the need to differentiate feigning from conversion disorder in clinical practice, particularly older psychiatrists (p=0.05, χ^2^= 15.8). Perhaps consequently, feigning was more confidently, or at least more frequently, addressed by the psychiatrists than neurologists. Even when feigning was not suspected, 7% said they would talk about it usually or always; when there was a degree of suspicion, 34% would; when confident, 54% would – far more than their neurological colleagues (p<0.001, χ^2^=30.4)

## Discussion

Psychiatrists strongly endorsed psychological models for conversion disorder, and the conversion model in particular, though many models were employed. They felt a psychiatric assessment was essential to the diagnosis of conversion disorder, and that they often disagreed with the diagnosis in neurologists’ referrals of putative conversion disorder. Most felt that a psychiatric formulation was supportive of, and many that it was necessary for, a diagnosis. Though few thought of conversion disorder as feigning, most saw these as overlapping concepts and they felt more comfortable discussing this than the neurologists previously surveyed.

The differences from the neurologists were perhaps less striking than might have been expected, given the divergent stereotypes with which the specialties are portrayed. There were notable similarities: the psychiatrists also saw conversion disorder as psychogenic rather than neurological; they also largely saw conversion disorder and feigning as overlapping but not the same; they also did not freely talk about feigning when they suspected it. Yet many differences, of degree, stood out: psychiatrists were stronger in their support of psychogenesis; less likely to think of deception when they thought of a memorable case; more confident in their ability to understand and formulate a patient psychosocially. But perhaps most striking were their views on the role of psychiatrists in diagnosis, and on the diagnostic relationship with neurology. Psychiatrists felt they were essential to the diagnostic process: they reported quite frequently sending patients back to neurologists because they believed the neurologist had got the diagnosis of conversion disorder wrong, a result which evokes psychiatrist Eliot Slater’s infamous claim that the diagnosis of “hysteria” represented systematic misdiagnosis by incompetent neurologists.^13^ Widespread psychiatric disagreement with neurologists’ diagnoses of conversion disorder has been reported more recently than that, from the neurologist perspective,^6^ and not only by us.^14^ But though from the point of view of neurologists this disagreement appears to be psychiatric error,^14^ it appears just as clearly, from these psychiatrists’ perspective, to be error by neurologists. Psychiatrists reported they were sending patients back not because they had overlooked conversion disorder, as neurologists strongly believed,^14^ but because they did not think conversion disorder was the right psychiatric diagnosis. Moreover, psychiatrists reported they would not, in general, accept the diagnosis without the presence of a formulation. Diagnoses made on neurological grounds, however confidently, were more likely to be rejected than accepted as conversion disorder, and a psychiatric formulation was clearly seen as diagnostic proof when there was neurological doubt. In both questions, large minorities thought a neurologist’s opinion could be decisive – but not all neurologists’ opinions.

Though the validity of the DSM-5 diagnostic criteria was not the subject of this survey directly, these results appear to raise significant doubts about psychiatric support for them. As this is the first survey of its kind that we are aware of, and the DSM field testing did not include conversion disorder,^15^ psychiatrists’ views are unlikely to have been taken into account in any systematic way. The new criteria are commonly interpreted as saying the diagnosis is now “inclusionary”, on the basis of its neurological signs alone,^16^ but this survey suggests psychiatrists do not agree. As they usually do not feel confident in diagnosing conversion disorder themselves, and do not feel confident that a neurologist can do so alone, this suggests support for the “old” two-step process, with assessment by a neurologist followed by diagnosis by a psychiatrist. Psychiatrists may, of course, simply be being conservative in this – though there was no association of these attitudes with age or experience, the changes are relatively new and the respondents may not have been familiar with them. As a group with a particular interest in neuropsychiatry, however, and who are seeing patients with conversion disorder with some regularity this seems less likely. Perhaps they are merely “protecting their turf”, and resisting further inroads into their prerogatives? Their reported diagnostic disagreements suggest they at least believe they have clinical reasons for doing so. Some of these reasons may be the less controversial role of psychiatrists in management of conversion disorder, and the importance of psychiatric formulation to that end^17^ – perhaps one interpretation of their response is that a diagnosis without formulation may be possible, but unwise.

Psychiatrists seem to have embraced some recent changes more than others. The term “Functional Neurological Disorder” is now the most frequently used diagnosis with patients but most still explain this in terms of psychogenic aetiology, notably as a “reaction to stress”. Though they perhaps did not differ starkly from neurologists on their attitudes to feigning, psychiatrists appeared less troubled by it and more willing to see it as part of a spectrum, always present.^18^ This suggests that the other major change in DSM-5 – dropping the requirement to specifically exclude feigning – would be supported by these respondents; indeed, their relative ease may suggest they have already adopted the change. On the other hand, it might also suggest that the two-stage process would be preferred, at least when feigning was a significant element, as the psychiatrists appear to feel much more comfortable discussing feigning than their neurological colleagues.

## Limitations

Any survey inherently introduces bias in shaping the way that answers are obtained, limiting the choices to predefined criteria and suggesting answers which may not have otherwise been considered. The use of multiple-choice questions may force the respondent to choose an option which may not exactly align with their beliefs; however, we used free text questions when possible to allow the individual to propose other ideas, most obviously in the “memorable case”. As no definition was given for the term “conversion disorder”, respondents will have depended on their own understanding of the condition, and the term “conversion” may have impelled respondents towards traditional interpretations; however, as the official psychiatric term for the disorder, this is less likely to have introduced any novelty than it was for the neurologists, and the provision of a definition could have introduced even more significant bias. Equally, terms such as “dissociation” and “psychogenesis” are notably ambiguous,^10^,^19^ which limits our interpretation of respondents’ views on these, but an attempt at acceptable definitions for these is an undertaking beyond the scope of this survey and would introduce bias of its own.

The use of an internet-based survey distribution for sampling may have also introduced bias, favouring younger or urban respondents. It probably contributed to the markedly reduced response rate compared with our paper survey of the neurologists, so the sample would inevitably be more restricted and more biased, presumably to those with the keenest interest, and perhaps the strongest opinions; the reduced response may also reflect a relative lack of interest within the specialty. All were drawn from groups defined by an interest in neuropsychiatry, though most did not work as neuropsychiatrists – and at median 4 cases per year, were not seeing very large numbers of patients with conversion disorder. While, on the one hand, this broadens the generalisability of these views beyond specialists, it also dilutes any claim to specific expertise in the area.

We sampled two countries, which clearly strengthens validity and generalisability, though those two countries are strongly linked by national and medical culture – and there were very few response differences between the two samples. By contrast, however, the survey of neurologists was of UK consultants only, so the comparison is limited thereby, as well as by elements of the survey process, though these did not appear to materially affect the neurologists’ responses.^20^ That survey was conducted in 2009, some 10 years prior to this one, so neurologists’ attitudes may well have changed, and that survey should be repeated.

Above all, these are survey data, and reflect what respondents think or remember about their behaviour, and their actual behaviour, for example, of disagreement with neurologists, may be very different.

## Conclusion

Psychiatrists believe conversion disorder to be psychogenic and to overlap with feigning. They believe psychiatrists are essential for the diagnostic process and would not usually support a diagnosis without a psychiatric formulation.

## Data Sharing Statement

No additional data are available.
